# Draft Genome Sequence of Acinetobacter calcoaceticus Strain P23, a Plant Growth-Promoting Bacterium of Duckweed

**DOI:** 10.1128/genomeA.00026-15

**Published:** 2015-02-26

**Authors:** Masayuki Sugawara, Akira Hosoyama, Atsushi Yamazoe, Masaaki Morikawa

**Affiliations:** a Division of Biosphere Science, Graduate School of Environmental Science, Hokkaido University, Sapporo, Japan; b Department of Green Innovation, Advanced Low Carbon Technology Research and Development Program (ALCA), Japan Science and Technology Agency (JST), Tokyo, Japan; c Biological Resource Center, National Institute of Technology and Evaluation, Tokyo, Japan

## Abstract

Acinetobacter calcoaceticus strain P23 is a plant growth-promoting bacterium, which was isolated from the surface of duckweed. We report here the draft genome sequence of strain P23. The genome data will serve as a valuable reference for understanding the molecular mechanism of plant growth promotion in aquatic plants.

## GENOME ANNOUNCEMENT

Acinetobacter calcoaceticus strain P23 was isolated as a phenol-degrading bacterium from the surface of a species of duckweed (Lemna aoukikusa) grown in the Hokkaido University Botanical Garden, Japan (1). It was previously observed that the bacterium colonizes on root surfaces and promotes the growth of duckweeds, including L. aoukikusa and Lemna minor (1, 2). The strain also increases the chlorophyll content not only in monocot duckweed but also dicot lettuce (Lactuca sativa) in a hydroponic culture (2). However, the molecular mechanism of the plant growth promotion by P23 remains unknown, and there is also quite limited information available on the interactions between microorganisms and aquatic plants, including duckweed. In this study, draft genome sequencing analysis was conducted to shed light on the molecular mechanism of plant growth promotion by A. calcoaceticus P23.

The genome of strain P23 was sequenced by single-end sequencing with 454 GS FLX+ (Roche, Basel, Switzerland) and paired-end sequencing with the Illumina HiSeq 1000 sequencing system (Illumina, San Diego, CA, USA). Two different types of libraries were constructed for sequencing, a standard library (600 to 700 bp) for GS FLX+ and a paired-end library (400 bp) for HiSeq 1000. A total of 60,992,398 base sequences (96,525 reads) were obtained, with a depth of 15-fold genome coverage, by the GS FLX+ system, and 591,774,320-base paired-end sequences (5,957,534 reads) were obtained, with a depth of 146-fold genome coverage, by the HiSeq 1000 system. The assembly was performed using the Newbler version 2.6 software (Roche). The assembled draft genome sequence was annotated using the xBASE bacterial genome annotation service (3) in reference to the genome of A. calcoaceticus PHEA-2 (4).

The P23 draft genome, which is 4,058,823 bp in length (38.5% G+C content), includes 3,730 protein-coding sequences (CDS), 3 rRNA genes, and 61 tRNA genes distributed in a total of 44 contigs (>100 bp). The complete set of genes involved in the phenol degradation pathway, *mphRKLMNOP* (P23_2971 through P23_2977) and *catMBCAIJFD* (P23_0869 through P23_0876), was predicted to have high homology (>95% identity by BLASTp) to that of the phenol-degrading bacterium A. calcoaceticus PHEA-2 (4). However, the genes involved in the activities of general plant growth-promoting traits, such as nitrogen fixation, indole-3-acetic acid biosynthesis, and 1-aminocyclopropane-1-carboxylate deaminase, have not been predicted in the genome. The genome sequence of P23 will serve as a valuable reference for understanding the plant growth-promoting factors of aquatic plants.

### Nucleotide sequence accession numbers.

The draft genome sequence of A. calcoaceticus P23 has been deposited at DDBJ/EMBL/GenBank under the accession numbers BBQU01000001 through BBQU01000044.
